#  A Pilot Study of Polymorphism of Adrenergic Beta-2 Receptor and Mild Asthma: A Clinical and Pharmacogenetic Study

**Published:** 2013

**Authors:** Parisa Adimi Naghan, Fanak Fahimi, Seyed Alireza Nadji, Nima Naderi, Fatemeh Soleimani, Mohammad Reza Masjedi

**Affiliations:** a *Chronic Respiratory Disease Research Center, NRITLD, Masih Daneshvari Hospital, Shahid Beheshti University of Medical Sciences, Tehran, Iran. *; b *Clinical Pharmacy Department, School of Pharmacy, Shahid Beheshti University of Medical Sciences, Tehran, Iran. *; c *Pharmaceutical Care Department, Chronic Respiratory Disease Research Center, NRILTD, Masih Daneshvari Hospital, Shahid Beheshti University of Medical Sciences, Tehran, Iran.*; d *Virology Research Center, NRITLD, Masih Daneshvari Hospital, Shahid Beheshti University of Medical Sciences, Tehran, Iran. *; e *Neuroscience Research Center, Shahid Beheshti University of Medical Sciences, Tehran, Iran.*; f *Toxicology Department, School of Pharmacy, Shahid Beheshti University of Medical Sciences, Tehran, Iran.*; g *Chronic Respiratory Disease Research Center, NRITLD, Masih Daneshvari Hospital, Shahid Beheshti University of Medical Sciences, Tehran, Iran. *

**Keywords:** Beta-adrenoceptor, Polymorphism, Asthma, Clinical study

## Abstract

Glycine allele at codon 16 has previously been associated with the increase in asthma severity, bronchial hyperresponsiveness and also the increase in inhaled corticosteroid dependence. This study was designed to evaluate the genetic alleles in mild asthma.

Thirty-four patients with diagnosis of mild asthma (FEV_1 _≥ 80%, positive methacholine test) and body mass index (BMI ≤ 30 Kg/m^2^) were included in the study. They could only use short acting beta-2 agonists for asthma control. Smoking, infection, occupational sensitizers’ exposure, gastroesophageal reflux, diabetes mellitus and heart failure were also considered as exclusion criteria. All patients were genotyped at 16^th^ and 27^th^ codons.

Among all, 20 (58.8%) Arg/Gly, 14 (41.2%) Arg/Arg and no Gly/Gly genotype were detected at codon 16. Genotyping at codon 27 revealed 2 (5.9%) Glu/Glu, 13 (38.2%) Glu/Gln and 18 Gln/Gln (52.9%).

Based on the obtained results, Arg/Gly mutation had a higher rate among the studied subjects compared to Arg/Arg polymorphism. This is a pilot study which shows a probable usefulness of genotyping for predicting of asthma severity.

## Introduction

Asthma is a chronic polygenic disease. The total cost per patient is estimated around 1,000 Euros yearly for patients with mild persistent asthma (1). 

Detection and diagnosis of asthmatic patients at risk of debility or death have always been a challenge (2). In some cases of near-fatal asthma, decreased function of *β*_2 _adrenergic receptors has been proposed (3) and thus, a hypothesis has suggested a defect in *β*_2_ adrenergic receptor to be responsible for pathogenesis of asthma (4). During the recent years, there have been reports regarding exacerbation of asthma as the result of long term use of *β*_2_ adrenergic agonists (5, 6). Based on the above-mentioned facts, we, along with a few other researchers, believe that the severity of asthma might be related to *β*_2_ adrenergic receptor genotype (7). In addition, a study by Drysdale on 13 polymorphisms revealed a huge diversion in distribution of some haplotypes between Caucasian, African-American, Asian and Hispanic-Latino (8). Such a difference was observed in some other studies which described ethnic-specific pharmacogenetic differences that could change the response of individuals to *β*_2_ adrenergic agonists (9-10).

In addition, our study would give us a basic view of Iranian mild asthmatic patients’ polymorphisms in *β*_2_ adrenoceptor gene. This pilot could benefit future studies as the first of its kind. The gene encoding this receptor is located on the short arm of chromosome 5 (11) and encodes one of the seven-transmembrane families of receptors that is coupled to the G protein and is expressed in various cell types like smooth muscle cells, neutrophils, eosinophils, macrophages and epithelial cells (12). Expression of *β*_2_ adrenergic receptors and their coupling are mediated through a dynamic process with a negative feedback cycle regulated in a way that in case of prolonged exposure to agonists or pre-inflammatory cytokines, down-regulation of receptors and a subsequent decreased response occur (13). In case of exposure to glucocorticoids, up-regulation of receptors occurs (14-15). There are 9 points in this gene that may undergo mutation (16). So far, 6 different types of polymorphisms have been detected (17), out of which, the arginine-to-glycine substitution at codon 16 and substitution of glutamic acid for glutamine at codon 27 are more common among the Caucasian population (16). The two above-mentioned substitutions along with the substitution of Threonine for Isoleucine at codon 164 have been shown to affect the function of receptor in *in-vitro *studies (2). *β*_2_ adrenoceptor agonists cause the dilation of airways and therefore, are indicated for the treatment of asthma (18). Several studies have discussed possible drug-related changes in *β*_2_ adrenergic receptors or signal transduction in cells that can control the disease. For example, in a study, polymorphism of *β*_2_ adrenergic receptors resulted in down regulation of them (19). The expression of Gln27 has been associated with hyperresponsiveness of airways in another study (20).

This study aimed at evaluating *β*_2_ adrenergic receptor polymorphism and its correlation with mild asthma in Iranian patients.

## Experimental

The study was conducted according to the ethical guidelines of Shahid Beheshti University of Medical Sciences for human studies and approved by the ethics committee of the university.

Patients with diagnosis of mild asthma (FEV_1_ ≥ 80% of predicted value, and positive methacholine test) who referred to the pulmonary clinic of Masih Daneshvari Hospital with the following inclusion criteria were entered into the study.

Cases with a history of gastroesophageal reflux, diabetes mellitus, heart failure, chronic obstructive pulmonary disease COPD, Churg-Strauss syndrome, bronchitis obesity (BMI > 30 Kg/m^2^), smoking or infections within one month prior to the study procedure or exposure to occupational sensitizers were excluded from the study. In addition, those who took medications which causes cough or exacerbate asthma condition, *i.e*. angiotensin converting enzyme inhibitors (ACEIs), nonsteroidal anti-inflammatory drugs (NSAIDs) and *β*-blockers, were not included in the study either. They could only use short acting beta2 agonists for asthma control.

Respiratory parameters of FEV_1_, FEV_1_/FVC were measured. Blood samples were collected and stored in -20°C for genotyping analysis.

*Molecular procedure*

*Genome extraction and PCR method*

**Figure 1:**
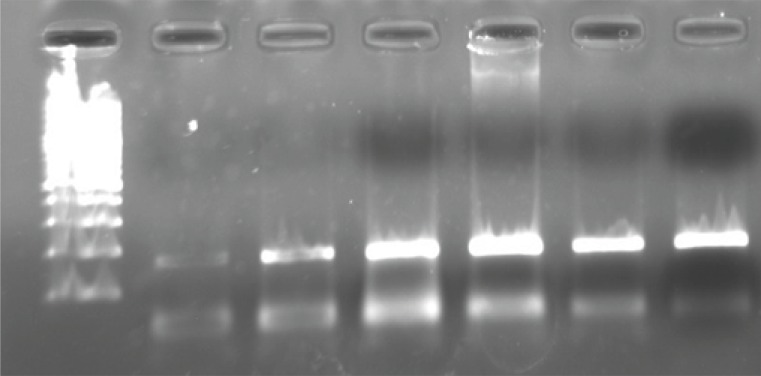
100 bp Molecular ladder is on the left side. PCR product bands stand at 168 bp

Genomic DNA was extracted from peripheral blood obtained using phenol chloroform method (21). The 2AR genotypes were determined by primer-induced restriction site assay. The primers were selected according to a previous study by Martinez *et al*. (22) and were 5’-GCCTTCTTGCTGGCACCCCAT-3’ and 5’-CAGACGCTCGAACTTGGCCATG-3’. Then, a PCR product which included the regions of *β*_2_AR-16 and *β*_2_AR-27 polymorphisms was generated. PCR reactions were carried out in a volume of 50 μL containing 50 ng of genomic DNA, 10 mM Tris-HCl (pH 8.3), 50 mM KCl, 1.5 mM MgCl_2_, 1.5 U of Taq polymerase, 0.2 mM of each deoxynucleotide triphosphate and 30 pm of each primer. Temperature cycling was 94°C for 60 s, 60°C for 60 s and 72°C for 60 sec for 44 cycles and then a final extension for 7 min at 72°C. The size of generated PCR product was 168 bp (Figure 1).

*Polymorphism detection*

**Figure 2:**
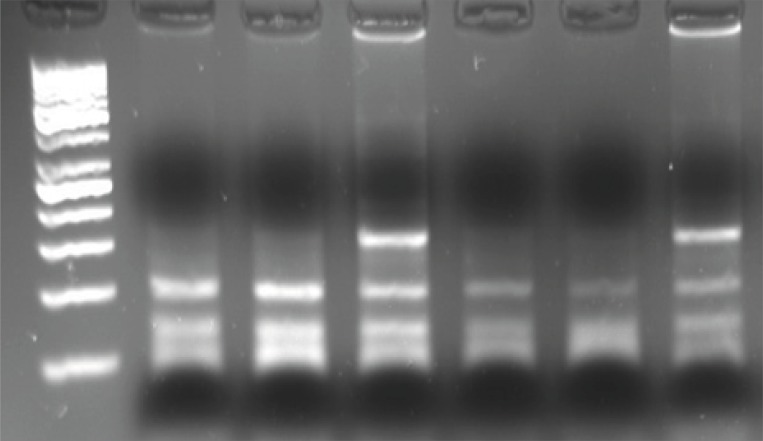
Identification of a polymorphism in amino acid 27 of beta2-adrenoceptor. Homozygotes for the glutamine 27 allele are in lanes 1, 2, 4 and 5. Gln/Glu heterozygotes are in lanes 3 and 6

For detection of *β*_2_AR polymorphism, 15 μL of PCR product was digested with 1 U of Nco. (New England Biolabs), 2 μL PCR water, 2 μL of 50 mM NaCl, 10 mM Tris-HCl, 10 mM MgCl_2_ and 1 mM dithiothreitol (pH 7.9) at 37°C for 12 h. Nco. cuts 22 bp from the 3’-end of both alleles and 18 bp from the 5’- end of the Gly-16 allele. The restriction digests were electrophoresed on 3% agarose gels and visualized with ethidium bromide staining and gel documentation. The Gln27Glu polymorphism was identified in the second restriction digest using another aliquot of the same PCR product. Twelve μL of PCR product was digested with 2 U of Bbv. (New England Biolabs), 5 μL PCR water, in 2 μL of 50 mM NaCl, 10 mM of Tris-HCl, 10 mM of MgCl_2_ and 1 mM of dithiothreitol (pH = 7.9) at 37°C for 12 h. Bbv digests the Gln-27 allele to produce 105 and 63-bp fragments which are separated from uncut Glu-27 alleles on 3% agarose gels (Figure 2).

## Results

Genotypes of 34 patients with mild asthma were evaluated in terms of polymorphisms at codons 16 and 27. Table 1 shows the demographics data and genotypic characteristics of patients. The mean ± SD age of patients was 29.97 ± 8.23 years (range = 16-52 years). Male to female ratio was 14/20. 

**Table 1:** Polymorphisms in codon 16 and 27 of mild asthmatic patients

**Codon**	**Genotype**	**Number (%)**
**Codon 16**	Arg/Arg	14 (41.2)
	Arg/Gly	20 (58.8)
	Gly/Gly	0 (0)
**Codon27**	Glu/Glu	2 (5.9)
	Glu/Gln	13 (38.2)
	Gln/GLn	18 (52.9)

Among all, 20 (58.8%) Arg/Gly, 14 (41.2%) Arg/Arg, and no Gly/Gly genotypes were detected at codon 16. Genotyping at codon 27 revealed 2 (5.9%) Glu/Glu, 13 (38.2%) Glu/Gln, and 18 Gln/Gln (52.9%). Frequency of Arg and Glu allele were estimated 70.6% and 25.75% respectively. Data of patients’ haplotypes in the study is shown in Table 2.

**Table 2:** The rate of *ß*_2_AR haplotypes in our study

**Codon/haplotype**	**Study results**
**Gly 16 - Glu 27**	9 (29.0)
**Gly 16 - Gln 27**	9 (29.0)
**Arg 16 - Gln 27**	9 (29.0)
**Arg 16 - Glu 27**	4 (12.9)
**Total**	31 (100)

## Discussion

B2AR (beta-2 adrenergic receptor) polymorphism has been studied and discussed by several studies in terms of asthma susceptibility, severity and responsiveness (2, 4, 7, 20, 22-25).

In our mild asthmatic patients, allele frequency of Arg allele (70.6%) was dominant compared to the frequency of Gly allele (29.4%). This finding is consistent with results of Liggette *et al. *study (26). The lower incidence of Gly allele in our patient population, might explain their mild symptoms. At 16^th^ codon, the presence of Arg allele seems to be protective in asthma severity in comparison with the other alleles resulted in milder asthma occurrence (27). In Liggette study, two groups of patients with and without nocturnal asthma were compared. The allele frequency of Gly16 was found to be higher in subjects with nocturnal asthma (80%) as compared with non-nocturnal asthmatic patients (52%). In addition, we could not find any patient with Gly/Gly similar to a study by Turki *et al*. (17). The study resulted in a small number of Gly homozygotes in non-nocturnal asthmatic patients, supporting the theory that Gly allele could be related to nocturnal asthma. In another study by Holloway *et al, *it was shown that the Gly16 polymorphism may play a role in the pathogenesis of asthma severity, but no significant relationship was found between the polymorphism at amino acid 27 and asthma or the asthma severity (28). Though, one of the reasons that our patients’ asthma severity was rated as mild, could be related to the fact that no patients with Gly/Gly polymorphism was among them.

Ramsey *et al. *could not find any association between 27Glu/Gln or Arg/Gln polymorphism and asthma (29).

On the other hand, in a study by Weir *et al. *which evaluated the polymorphisms at codones 16, 27, and 164 in 86 patients, B2AR polymorphism was not recognized a causative strong factor for asthma exacerbation. No significant difference between Gly allele frequencies was detected between patients with mild and moderate asthma. Gly16/Glu27 haplotype was more prevalent in mild asthmatic patients (2).

In general, it is clear that various host and environmental factors other than polymorphism can affect the severity of asthma; factors such as poor economic condition, lower level of education, concomitant diseases such as diabetes, heart failure, *etc *(30). For example, allergy was found as another significant predictor in a model derived from a retrospective logistic regression analysis, which proves that the positive history of allergy is a risk factor of asthma severity (31). But the dominant role of genetics should not be ignored.

This study had some limitations as well. Owing to the extensively limited inclusion criteria, we were not able to enroll more patients in the study. So, small sample size limits the power of the study to represent this data as a perfect model of Iranian asthmatic patients. Studies with a bigger sample size should be conducted to give more reliable data for general population of Iranian patients. As a first time population study, it would be better to include a group of non-asthmatic patients as control group. However, this was the first study in this realm conducted on asthmatic patients in Iran and paved the way for further investigations on such patients.
